# Superplastic Forging for Sialon-based Nanocomposite at Ultralow Temperature in the Electric Field

**DOI:** 10.1038/s41598-019-38830-1

**Published:** 2019-02-21

**Authors:** Junting Luo, Chenyang Xi, Yongfei Gu, Lili Zhang, Chunxiang Zhang, Yahong Xue, Riping Liu

**Affiliations:** 1 Education Ministry Key Laboratory of Advanced Forging & Stamping Technology and Science, Yanshan University, Qinhuangdao, 066004 China; 2 State Key Laboratory of Metastable Materials Science and Technology, School of Mechanical Engineering, Yanshan University, Qinhuangdao, 066004 China; 3 School of Art and Design, Yanshan University, Qinhuangdao, 066004 China

## Abstract

The utralow-temperature superplastic forging for sialon-based nanocomposites is reported for the first time. Sialon-based nanocomposites, with an average grain size smaller than 50 nm and 98.5% relative density, were prepared with nano-sized row powders by the spark plasma sintering (SPS) technique at a record ultralow sintering temperature of 1150 °C. An excellent gear is forged at the ultralow deformation temperature of 1200 °C with nanosized grains without any cracking. The maximum strain rate achieved is over 10^−1^ s^−1^, and a compression strain is more than 0.9. The practical application for superplastic forming of nitrogen ceramics is much more difficult than that for oxide ceramics because of the high deformation temperature and low strain rates. The present findings present a bright prospect for the near-net-shape superplastic forming of nitrogen ceramics.

## Introduction

As high-temperature structural materials, silicon nitride-based ceramics are the excellent mechanical properties, high melting temperature, low density, high elastic modulus and strength, and good resistance to creep, wear and oxidation. The intrinsic brittleness and hardness of silicon nitride-based ceramics, however, make it difficult and costly to machine them into complex-shaped components^1^. Now things are changing since Chen^2^ and Wakai^3^ independently discovered superplasticity of silicon nitride- based ceramics in 1990.

To enhance the superplasticity and reduce the deformation temperature of Si_3_N_4_-based ceramics, the grain size of deformed materials must be refrained. A number of researchers have demonstrated superplasticity in several silicon nitride ceramics by applying the transient liquid phase^4,5^, using ultrafine β-phase powders^6,7^, or by adding secondary phases into Si_3_N_4_ to refine the microstructure^8–10^. Efforts have been also made to use special sintering technologies, such as sparking plasma sintering (SPS)^11^, and the grain size of superplastic Si_3_N_4_-based ceramics is submicrometer level. The grain size of sintered bodies usually is bigger than 0.2μm, so that the superplastic deformation temperature is higher than 1600 °C and typically strain rate is lower than 10^−4^s^−1^ ^12,13^. The nano-structured Si_3_N_4_-based ceramic has been reported by Xu^14–19^ by using SPS technology. However, the superplastic deformation temperature is higher than 1550 °C.

The low-temperature and high-strain-rate superplastic forming of oxide ceramics nanocomposites were reported by Zhan^20^ *et al*. and the application research has spread widely^21^. The practical application for superplastic forming of nitrogen ceramics is much more difficult than that for oxide ceramics because the high deformation temperature and low strain rates. By using four kinds of nano-sized powders and applying SPS technology, the authors have developed superplastic sialon-based nanocomposite with 50 nm grain size and investigated its formidable deformation capability of superplastic forging at low temperature of 1200 °C and high strain rate over 10^−2^ s^−1^.

## Experimental

The starting powders were amorphous nano-sized Si_3_N_4_ powders (China Northeast Ultrafine Powders Manufacture Co., Ltd.), nano-sized AlN powders (China Henfei Kaier Co., Ltd.). The average particle size of Si_3_N_4_ powders is 18 nm and AlN powder is 50 nm. The sintering assistants used were high-purity nano-sized Al_2_O_3_ and Y_2_O_3_ powders prepared by polymer network lamide gel method with a average grain size of 20 nm^22^. The powders (72 wt%Si_3_N_4_, 14 wt%AlN, 4 wt%Y_2_O_3_ and 10 wt%Al_2_O_3_) is wet milled in anhydrous acetone medium for 24 hours, and the grinding balls and tanks used are made of Sialon ceramics. Firstly, the powder is dried, then sintered in SPS device, SPS-3.20MK-IV with Nitrogen protection. The powder was loaded into a cylindrical high strength graphite die with an inner diameter of 20 mm. Samples and dies are heated by pulsed DC power supply. The heating rate is 300 °C·min^−1^ before 600 °C, and when the temperature reaches 600 °C, the infrared temperature measurement begins. When the temperature exceeds 600 °C, the heating rate is still 300 °C·min^−1^, and the axial pressure during sintering is 30 MPa. In the temperature range of 1000–1800 °C, the cooling rate is set to 400 °C·min^−1^. The powder mixture was sintered in nitrogen atmosphere at 1150 °C and 1200 °C for 2 min holding time respectively. The temperature was raised from 600 °C to specify the sintering temperature in 2 min.

In order to compare the sintering process, samples were also sintered by hot pressing sintering (HPS) using ZRY-120 furnace. When the temperature rises to 1700 °C, the heating time is 120 minutes, and the holding time at 1700 °C is 30 minutes. The same 30 MPa axial pressure is used during hot pressing sintering. The sintering curves of temperature vs. displacement are recorded by test system of two equipments.

The superplastic deformation was carried out in the SPS apparatus. The sintered compact cylindrical sample (diameter 20 mm, height 10 mm) was loaded into the graphite die with gear cavity, and then extruded, and the loading pressure during the deformation process was 20 MPa. The deformation temperature is 1200 °C. The gear cavity was formed by radical flow of nano-structured ceramic material.

The strain is expressed by ln(1-ΔL/L_0_), in which ΔL and L_0_ represent the deformation of the sample and the original height of the sample, respectively. The strain rate is the derivative of strain to time, which is consistent with the traditional concept.

The relative density was measured by Archimedes law. The phase present in the sintered ceramics were determined by X-ray diffraction (D-max-2500), using monochromatic CuKα radiation. Microstructure of the sintered samples was examined in a scanning electron microscopy (AMRAY-1000B). The grain boundary was observed by high resolution transmission electron microscopy (H-800), in order to obtain the image of amorphous films.

## Results and Discussion

**Figure 1:**
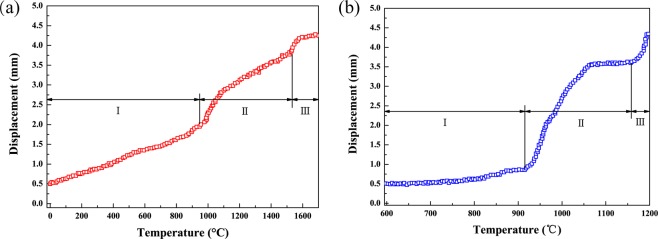
The punch displacement vs. sintering temperature for sailon-based ceramic in a conventional HP furnace and in an SPS apparatus respectively. There are three stages both for two sintering technologies, namely temperature raising, sintering and superplastic deformation. (**a**) The curve of punch displacement vs. sintering temperature for HPS, with heating rate of 40 °C/min and 20 MPa constant pressure. (**b**) The curve of punch displacement vs. sintering temperature for SPS, with heating rate of 300 °C/min and 20 MPa constant pressure. Obviously, the initial temperature of superplasticity in the electric field is much lower than the temperature at the time of hot pressing.

The punch displacement data obtained plotted vs. sintering temperature for sailon-based ceramic in a ZRY-120 furnace and in an SPS apparatus are shown in Fig. 1, respectively. There are three stages both for two sintering technologies, namely temperature raising, sintering and superplastic deformation.

For two sintering techniques, the beginning sintering temperature are all at about 950 °C, but there is much more difference for sintering rate and the powder densification is more slower for hot pressing sintering than for spark plasma sintering. When the temperature approaches 1600 °C for hot pressing sintering, materials basic density and relative density up to 98.2%, and then enter the superplastic forming stage. However, the densification is very quickly for spark plasma sintering. When the temperature reaches 1150 °C, materials already is more compact with 98.5% relative density, and begins into superplastic forming stage, which is 450 °C much lower than that for hot pressing sintering.

**Figure 2:**
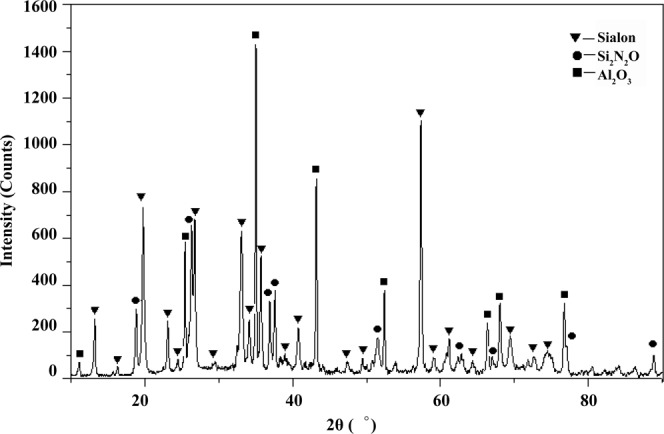
Phase compositions of Sialon-based composite sintered at 1150 °C analysised by X-ray diffraction. There are three phases, including Sialon, Si_2_N_2_O and Al_2_O_3_. The relative volume content of Sialon phase is more than 80% and the relative volume content of Si_2_N_2_O and Al_2_O_3_ is less than 20%.

Phase compositions of sialon-based composite sintered at 1150 °C are analyzed by X-ray diffraction. The results are shown in Fig. 2. There are three phases, including sialon, Si_2_N_2_O and Al_2_O_3_. The relative volume content of Sialon phase is more than 80% and the relative volume content of Si_2_N_2_O and Al_2_O_3_ is less than 20%, which indicates that the partial Al_2_O_3_ is not solid solution complete because of the ultralow sintering temperature.

**Figure 3:**
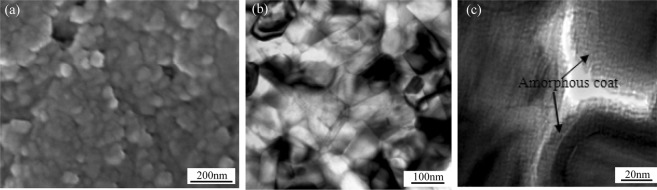
Scanning electron microscopy and transmission electron microscopy images of Sialon-based ceramic compacts before deformation in an electric field. (**a**) SEM microstructure before deformation. It consists of an equixial grain phase with about 50 nm grain size. (**b**) TEM microstructure before it is deformed. (**c**) The clear grain boundary is presented and there is amorphous coat with about 5~10 nm thickness at the grain interface.

The scanning electron and transmission electron microscope images of sialon-based ceramic compact before being deformed in an electric field are shown in Fig. 3. It consists of equixial and ultrafine grains with about 50 nm grain size. The clear grain boundary is presented for Fig. 3c and there is amorphous coat with about 5~10 nm thickness at the grain interface, which is considered to be one of the major causes of nitride ceramic with superplasticity^23,24^. The amorphous coat is usually formed by chemical reaction from (1) to (5)^9^.

1 $${{\rm{3Y}}}_{{\rm{2}}}{{\rm{O}}}_{{\rm{3}}}({\rm{s}})+{{\rm{Si}}}_{{\rm{3}}}{{\rm{N}}}_{{\rm{4}}}({\rm{s}})={{\rm{3Y}}}_{{\rm{2}}}{{\rm{O}}}_{{\rm{3}}}{{\rm{gSi}}}_{{\rm{3}}}{{\rm{N}}}_{{\rm{4}}}({\rm{s}})$$

2 $${{\rm{2Si}}}_{{\rm{3}}}{{\rm{N}}}_{{\rm{4}}}({\rm{s}})+{{\rm{3Y}}}_{{\rm{2}}}{{\rm{O}}}_{{\rm{3}}}{{\rm{gSi}}}_{{\rm{3}}}{{\rm{N}}}_{{\rm{4}}}({\rm{s}})={{\rm{3Y}}}_{{\rm{2}}}{\rm{Si}}({{\rm{Si}}}_{{\rm{2}}}{{\rm{O}}}_{{\rm{3}}}{{\rm{N}}}_{{\rm{4}}})({\rm{l}})$$

3 $$\,{{\rm{Si}}}_{{\rm{3}}}{{\rm{N}}}_{{\rm{4}}}({\rm{s}})+{\rm{2}}\,{{\rm{Al}}}_{{\rm{2}}}{{\rm{O}}}_{{\rm{3}}}({\rm{s}})={\rm{4AlN}}({\rm{s}})+{{\rm{3SiO}}}_{{\rm{2}}}({\rm{s}})$$

4 $${{\rm{2Si}}}_{{\rm{3}}}{{\rm{N}}}_{{\rm{4}}}({\rm{s}})+{\rm{10AlN}}({\rm{s}})+{{\rm{4Al}}}_{{\rm{2}}}{{\rm{O}}}_{{\rm{3}}}({\rm{s}})={{\rm{3Si}}}_{{\rm{2}}}{{\rm{Al}}}_{{\rm{6}}}{{\rm{O}}}_{{\rm{4}}}{{\rm{N}}}_{{\rm{6}}}({\rm{l}})$$

5 $${{\rm{5Si}}}_{{\rm{3}}}{{\rm{N}}}_{{\rm{4}}}({\rm{s}})+{{\rm{21SiO}}}_{{\rm{2}}}({\rm{s}})+{{\rm{14Al}}}_{{\rm{2}}}{{\rm{O}}}_{{\rm{3}}}({\rm{s}})={{\rm{4Si}}}_{{\rm{9}}}{{\rm{Al}}}_{{\rm{7}}}{{\rm{O}}}_{{\rm{21}}}{{\rm{N}}}_{{\rm{5}}}({\rm{l}})$$

In the process of SPS, the grain boundary area is huge due to the role of nanocrystallization. The sintering auxiliaries dispersed on the grain boundaries are instantaneously melted by discharge plasma generated by high current, while the overall temperature of the material is very high, resulting in the decrease of the superplastic deformation temperature.

**Figure 4:**
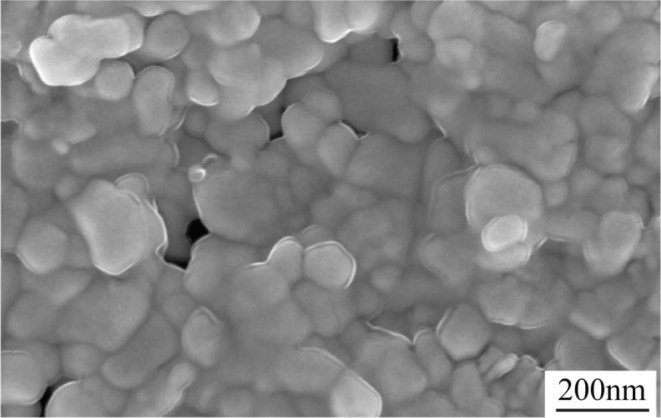
Scanning electron microscope images of Sialon-based ceramic compact after deformation in an electric field. It is typical non-equilibrium equixial grain topography. Some grains grow up to about 80 nm and the others keep stable with 50 nm.

The scanning electron microscope image of sialon-based ceramic compact after deformation in an electric field is shown in Fig. 4. It is typical non-equilibrium equixial grain topography. The most grains are not growth during the ultralow-temperature and high strain rate deformation, a handful of grains grows up to about 80 nm and the most others keep stable with 50 nm, which ensures the good superplasticity during forging^25^.

**Figure 5:**
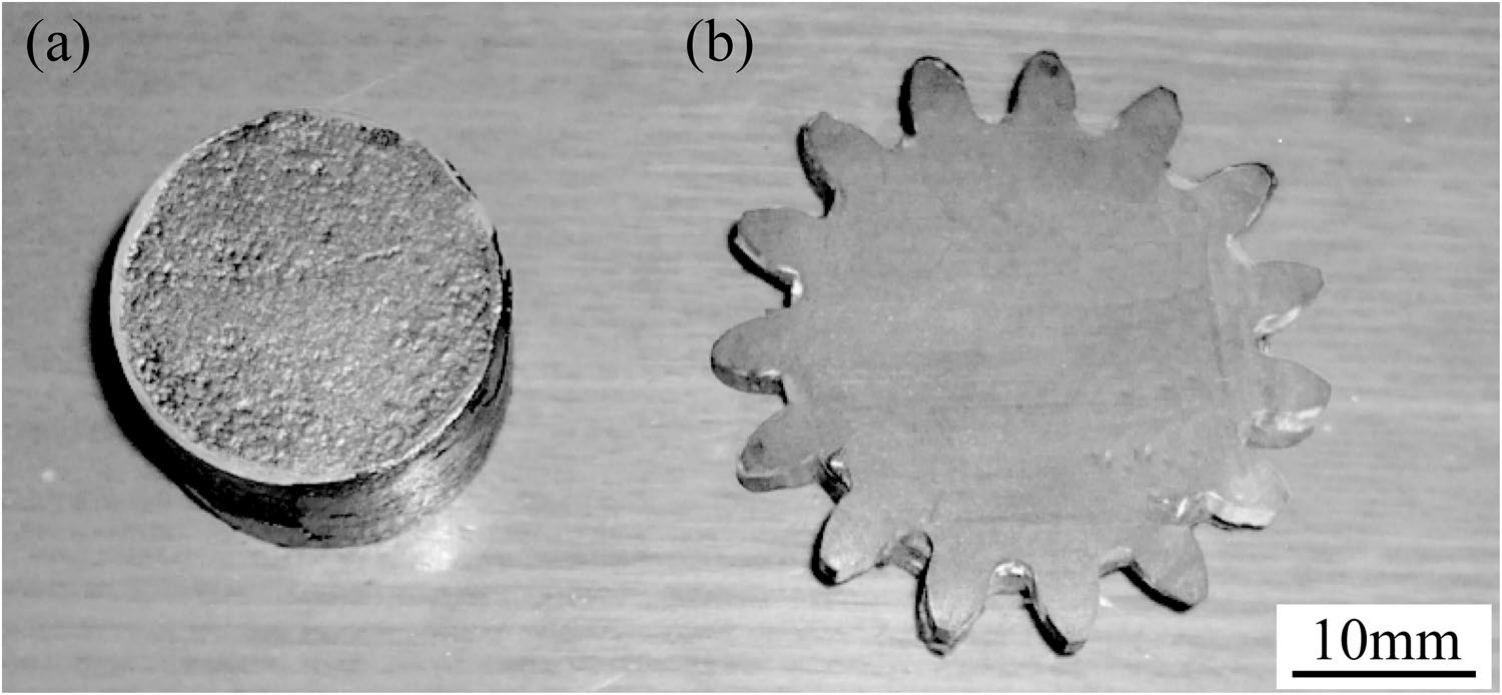
A photograph of a gear with 3.8 mm height forged using nano-structured sialon-based ceramic in a graphite die at 1200 °C under the action of an electric field is shown to the left. The shape of the deformed part is complex, the shape of the gear is sharp, and the surface finish is excellent. It is formed in 2 s with an axial pressure of 20 MPa. The billet (shown at right) is cylindrical (diameter 20 mm, height 10 mm).

The columnar blank before deforming is shown in Fig. 5(a), with 20 mm diameter, 10 mm height, and 98.5% relative density. A photograph of a gear forged is shown in the right of Fig. 5(b). The gear material is a nanostructured sialon-based ceramic formed under the action of an electric field (in an SPS device) in a graphite die at 1200 °C. The shape of the deformed part is complex, the shape of the gear is sharp, and the surface finish is excellent. It is formed in 2 s with an axial pressure of 20 MPa.

**Figure 6:**
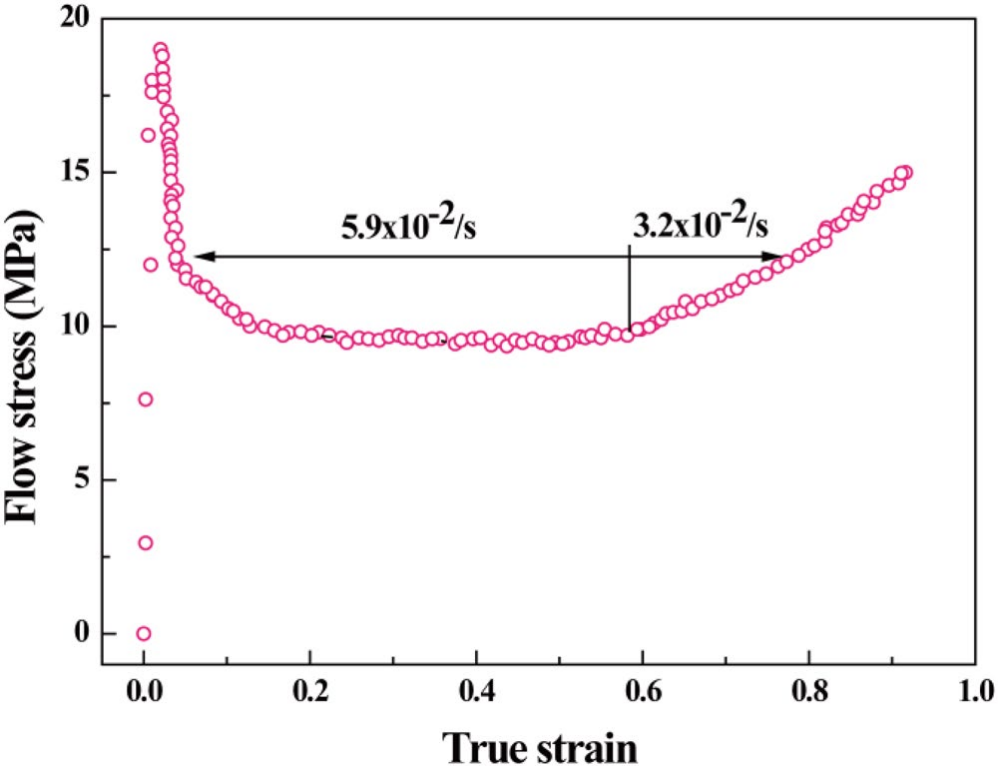
Spark-plasma-enhanced superplastic deformation behavior of sialon-based nanocomposite. The curve of true strain vs. flow stress for the superplastic forging of gear is under 1200 °C.

The curve of true strain vs. flow stress for the superplastic forging of gear at 1200 °C is shown in Fig. 6. The stress reaches a maximum value in the initial stage of deformation (less than 20 MPa), then rapidly decreases to about 10 MPa and keeps stable. In the final stages of tooth profile close to the full, the stress increased again to 15 MPa. The strain rate is about 5.9 × 10^−1^ s^−1^ when the true strain is less than 0.6 and about 3.2 × 10^−1^ s^−1^ when the true strain is more than 0.6.

Finally, superplastic forming technology for ceramic materials application is very magic and important. Sialon-based nanocomposite has been obtained and fine gear are forged by superplastic forming firstly at ultralow-temperature, which are pioneering significance for superplastic forming technology application of covalent ceramics. The superplastic deformation temperature is below the oxide ceramics and slightly higher than the deformation temperature of high temperature alloy, which changes the concept of low temperature superplastic forming only for the oxide ceramic with ionic bond. These results suggest that the superplastic near-net-shape forming technology with low-temperature and high-strain-rate has bright application prospect for various types of ceramics.
